# In Vitro Antibacterial, Anti-Adhesive and Anti-Biofilm Activities of *Krameria lappacea* (Dombey) Burdet & B.B. Simpson Root Extract against Methicillin-Resistant *Staphylococcus aureus* Strains

**DOI:** 10.3390/antibiotics10040428

**Published:** 2021-04-13

**Authors:** Carlo Genovese, Floriana D’Angeli, Francesco Bellia, Alfio Distefano, Mariarita Spampinato, Francesco Attanasio, Daria Nicolosi, Valentina Di Salvatore, Gianna Tempera, Debora Lo Furno, Giuliana Mannino, Fabio Milardo, Giovanni Li Volti

**Affiliations:** 1 Section of Microbiology, Department of Biomedical and Biotechnological Sciences, University of Catania, via Santa Sofia 97, 95123 Catania, Italy; dnicolosi@unict.it; 2 Department of Drug and Health Sciences, University of Catania, Viale Andrea Doria 6, 95125 Catania, Italy; 3 Nacture S.r.l, Spin-Off University of Catania, via Santa Sofia 97, 95123 Catania, Italy; tempera@unict.it; 4 Section of Biochemistry, Department of Biomedical and Biotechnological Sciences, University of Catania, via Santa Sofia 97, 95123 Catania, Italy; fdangeli@unict.it (F.D.); distalfio@gmail.com (A.D.); mariaritaspampinato93@gmail.com (M.S.); livolti@unict.it (G.L.V.); 5 Department of Human Sciences and Quality of Life Promotion, San Raffaele Roma Open University, via Val Cannuta 247, 00166 Rome, Italy; 6 Institute of Crystallography, National Research Council (CNR), Via Paolo Gaifami, 18, 95126 Catania, Italy; francesco.bellia@cnr.it (F.B.); francesco.attanasio@cnr.it (F.A.); 7 Section of General and Clinical Pathology and Oncology, Department of Biomedical and Biotechnological Sciences, University of Catania, via Santa Sofia 97, 95123 Catania, Italy; valentina.disalvatore@unict.it; 8 Section of Physiology, University of Catania, Department of Biomedical and Biotechnological Sciences, via Santa Sofia 97, 95123 Catania, Italy; lofurno@unict.it (D.L.F.); giuliana.mannino@unict.it (G.M.); 9 Herbalist Shop of Dr. Milardo Fabio, via Fonte 2, 96010 Melilli, Italy; fabiomilardo@gmail.com

**Keywords:** *Krameria lappacea*, methicillin-resistant *Staphylococcus aureus*, biofilm, adhesion and invasion, A549 cells, proanthocyanidins

## Abstract

Methicillin-resistant *Staphylococcus aureus* (MRSA) represents a serious threat to public health, due to its large variety of pathogenetic mechanisms. Accordingly, the present study aimed to investigate the anti-MRSA activities of *Krameria lappacea*, a medicinal plant native to South America. Through Ultra-High-Performance Liquid Chromatography coupled with High-Resolution Mass spectrometry, we analyzed the chemical composition of *Krameria lappacea* root extract (KLRE). The antibacterial activity of KLRE was determined by the broth microdilution method, also including the minimum biofilm inhibitory concentration and minimum biofilm eradication concentration. Besides, we evaluated the effect on adhesion and invasion of human lung carcinoma A549 cell line by MRSA strains. The obtained results revealed an interesting antimicrobial action of this extract, which efficiently inhibit the growth, biofilm formation, adhesion and invasion of MRSA strains. Furthermore, the chemical analysis revealed the presence in the extract of several flavonoid compounds and type-A and type-B proanthocyanidins, which are known for their anti-adhesive effects. Taken together, our findings showed an interesting antimicrobial activity of KLRE, giving an important contribution to the current knowledge on the biological activities of this plant.

## 1. Introduction

**Figure 1:**
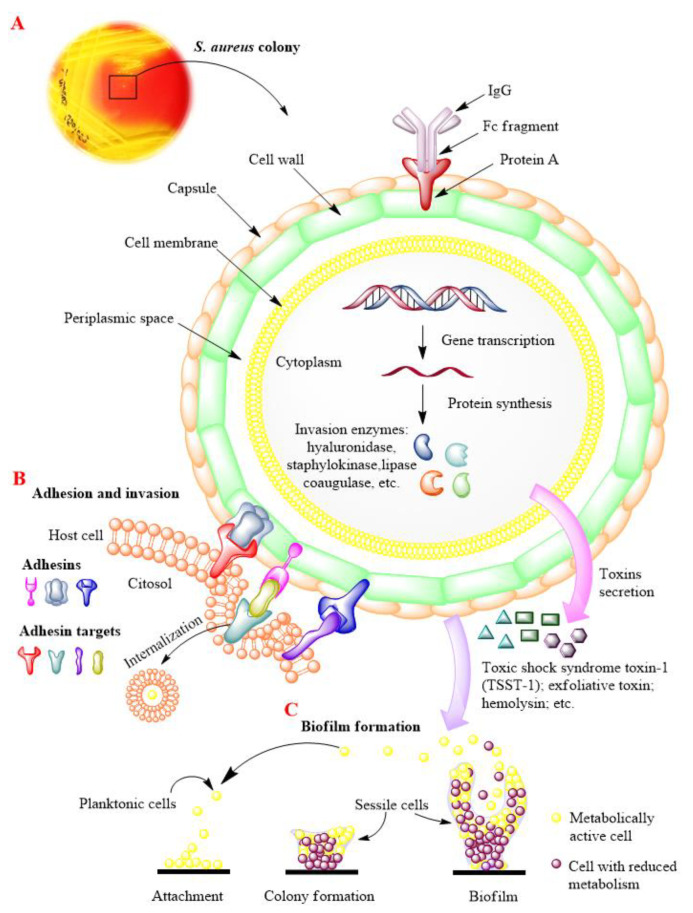
Schematic model of the virulence factors of *S. aureus*. (**A**). Cell structure of *S. aureus*. The cell-wall contains several proteins, including Protein A which binds the Fc fragment of immunoglobulin-G (IgG), blocking its phagocytic activity, and the adhesins, which mediate the adhesion and invasion of the bacterium to the host cell. Besides, *S. aureus* synthesizes different exoenzymes, which facilitate its dissemination and tissue invasion. This pathogen is also able to secrete a large variety of toxins, responsible for severe diseases. (**B**). Molecular mechanisms involved in the adhesion and invasion of *S. aureus* to host cell. The binding of surface adhesins with specific cellular receptors or with the component of extracellular matrix triggers a signalling cascade that culminates with bacterial internalization. (**C**). Biofilm formation stages. Favorable environment conditions promote the formation of biofilm, which consists of three different stages: attachment of free bacteria (planktonic cells) to the abiotic surface (e.g., medical devices); initial synthesis of biofilm and formation of bacterial colony inside the biofilm (sessile bacteria) and, finally, complete biofilm formation. The bacteria embedded in the biofilm can reduce their metabolic activity, becoming quiescent. Under specific environment signals, the sessile bacteria can switch to planktonic form, provoking reinfection.

Bacteria colonization of specific districts of the human body constitutes what is defined as the “human microbiome”. Among the symbiotic microbial species, it is possible to recognize *Staphylococcus aureus* (*S. aureus*), a Gram-positive bacterium, commonly present in the skin, skin glands, and mucosal membranes of healthy individuals [1,2,3]. As a commensal organism is generally harmless for the host. However, *S. aureus* possesses a high pathogenic potential thanks to a vast repertoire of virulence factors (Figure 1). Indeed, it produces a large variety of toxins, including the characteristic toxic shock syndrome toxin-1, able to trigger an abnormal immune response, provoking the homonym syndrome. Moreover, the surface of this microorganism is studded by over 20 proteins, such as the adhesins, which mediate the adhesion and invasion of the pathogen to host cells [4,5,6]. Besides, *S. aureus* is also endowed with an external capsule of a polysaccharidic nature, that allows it to escape from the phagocytic action of the immune cells [7,8]. This ability is also supported by the expression of a series of enzymes, including coagulase [9], lipase [10], hyaluronidase [11], staphylokinase [12], by which the bacterium can elude the host’s innate and adaptive immune response, promoting tissues invasion. A further evasion mechanism consists of biofilm production, a polysaccharidic matrix, which acts like a barrier protecting itself from the immune system and antibiotics action, thus favoring its persistence in the host [13].

Furthermore, in the clinical setting, this pathogen is feared for the presence of methicillin-resistant *S. aureus* (MRSA) strains, which make the related infections extremely difficult to treat. Such resistance occurs following the acquisition of the *mecA* gene, contained in mobile genetic elements known as staphylococcal cassette chromosome mec, by a susceptible strain [14,15]. This gene encodes for an altered penicillin-binding protein (PBP), the PBP2a, characterized by a lower affinity for the β-lactams [14]. Besides, MRSA strains are often resistant to other classes of antibiotics, including quinolones, aminoglycosides, and macrolides [16].

These pathogenic features clearly explain the prominent role of MRSA among the bacterial related community and hospital-acquired infections (CAI and HAI). A recent surveillance study, involving 890 hospitals overall, estimated a total of 622,390 infections, due to multidrug-resistant bacteria, among hospitalized patients, of which 104,572, equal to 17%, were acquired in the hospital setting. Interestingly, MRSA-related infections accounted for 52% of the cases, therefore ranking as the leading cause of HAIs [17]. Among MRSA-related HAIs, hospital-acquired pneumonia (HAP) and ventilator-associated pneumonia (VAP) assume particular relevance. Indeed, this pathogen is able to colonize the lower respiratory tract of patients afflicted with chronic pulmonary diseases or undergoing endotracheal intubation and mechanical ventilation. In these cases, the reduced host defenses facilitate the virulent role of *S. aureus*, resulting in the onset of MRSA-related pneumonia [18,19].

The increasingly limited availability of efficient therapies able to counteract the pulmonary complications induced by MRSA strains strongly encouraged the research of new molecules exerting antimicrobial activity. It is well established that natural extracts constitute an outstanding source of biologically active compounds, some of which have demonstrated antibacterial and anti-biofilm effects [20,21].

In this regard, *Krameria lappacea* (Dombey) Burdet & B. B. Simpson; (syn. *K. triandra* Ruiz et Pavon), a hemiparasitic plant, native to the Andean Mountains of Bolivia and Peru is endowed with benefic effects on human health. Indeed, *Krameria lappacea* (*K. lappacea*), also known as Rhatany, was traditionally used as a remedy, mainly for the treatment of gastrointestinal disorders [22]. Furthermore, its root possesses anti-inflammatory and anti-hemorrhagic properties, which promoted folk use in cases of stomach ailments, oropharyngeal inflammation, and excessive blood loss [23,24]. Nevertheless, to date, there are few and mostly dated studies focusing on the biological activity of *K. lappacea* root extract (KLRE). Specifically, the anti-inflammatory [25], antioxidant [26], photoprotective [24], and antimicrobial properties of KLRE have been documented. Concerning the latter aspect, Scholz and Rimpler, in 1989, reported the antibacterial effect of KLRE against *S. aureus* [27]. Moreover, a recent study revealed the antimicrobial effect of both ethyl acetate and methanol extracts of *K. lappacea* against Gram-positive bacterial strains, including *S. aureus* [28]. However, these pieces of evidence merely consist of MIC values. However, in this study, the authors mainly investigated the chemical composition of the extract. Specifically, they isolated and characterized the tannins of Rhatany root, in particular the proanthocyanidins [27].

Therefore, in our work, we proposed to fill data gaps, by examining more thoroughly the antimicrobial activity of this extract against one of the most relevant bacteria, affecting the population’s health. For this purpose, after preliminary screening of ten clinical isolates and one standard *S. aureus* strains, we selected the major biofilm producers, in order to analyze the ability of KLRE to inhibit the growth and the biofilm formation of these strains. Moreover, we evaluated the capacity of the extract to eradicate the biofilm previously formed by bacteria. Furthermore, given the pathogenic role played by *S. aureus* at the pulmonary level, we also investigated whether the KLRE was able to prevent the adhesion and invasion of the selected strains against the human lung carcinoma A549 cell line. Finally, we also performed the chemical analysis of the KLRE, by using an approach based on Ultra High-Performance Liquid Chromatography (UHPLC) coupled with High-Resolution Mass spectrometry (HRMS).

## 2. Results

### 2.1. Susceptibility of S. aureus Strains to Cefoxitin and Oxacillin

*S. aureus* clinical strains showed resistance to cefoxitin, with a diameter inhibition zone ≤21 mm. Clinical and Laboratory Standard Institute (CLSI) does not report any zone diameter breakpoints for oxacillin, since cefoxitin is tested as a surrogate for determining methicillin-resistance [29]. The standard strain was susceptible to cefoxitin, with an inhibition diameter of 31 mm (Table S1).

### 2.2. Detection of Macrolide-Lincosamide-Streptogramin B Phenotypes

All the *S. aureus* strains, except for *S. aureus* ATCC 6538 and MRSA 10, were resistant to both erythromycin and clindamycin. Besides, the resistant strains showed the constitutive macrolide–lincosamide–streptogramin B (cMLSB) phenotype (Table S1).

### 2.3. Detection of Penicillin-Binding Protein 2a (PBP2a)

The latex agglutination test allowed us to detect PBP2a extracted from bacterial strains. *S. aureus* clinical isolates determined a strong visible positive reaction. Conversely, *S. aureus* ATCC 6538 did not cause any agglutination. According to the manufacturer’s instructions, the methicillin-resistant strain *S. aureus* ATCC 43300 and the methicillin-susceptible strain *S. aureus* ATCC 29213 were used as positive and negative controls, respectively (Table S1).

### 2.4. Antibacterial Activity

KLRE and ciprofloxacin exerted a bactericidal activity since the Minimal Bactericidal Concentration (MBC)/Minimal Inhibitory Concentration (MIC) ratios were ≤4. The natural extract was able to inhibit the growth of all the tested *S. aureus* strains with a MIC value of 64.00 µg/mL. Conversely, ciprofloxacin showed MIC values ranging from 0.25 to 64.00 µg/mL (Table 1).

### 2.5. Biofilm Quantification Assay

**Figure 2:**
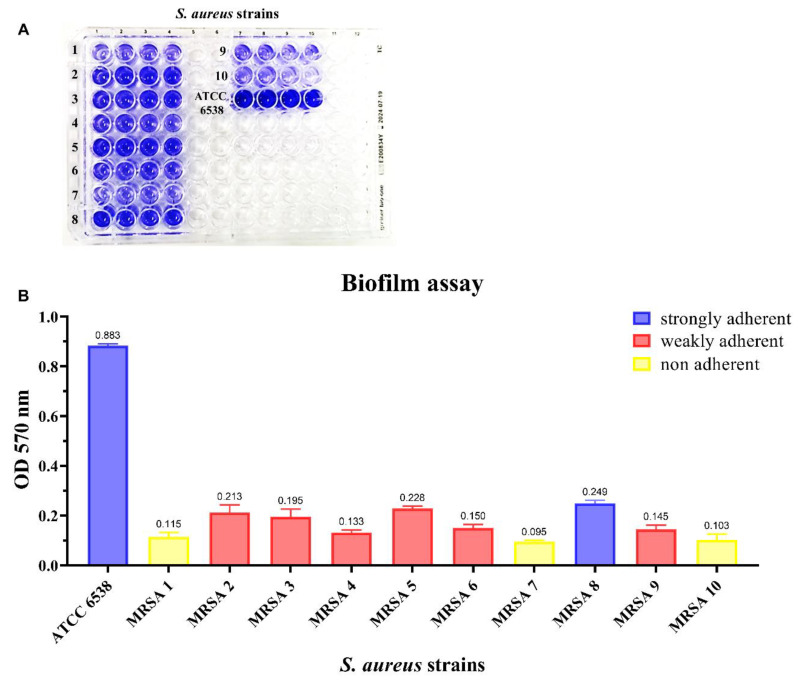
Biofilm quantification assay of methicillin-resistant *Staphylococcus aureus* strains. (**A).** Representative figure of biofilm quantification by crystal violet assay in 96-well microplate. (**B**). Quantitative analysis of biofilm formation. Bacteria were categorized as non-adherent (O.D. ≤ 0.120: yellow histograms), weakly adherent (O.D. > 0.120: red histograms) and strongly adherent (O.D. > 0.240: blue histograms) [30]. In the x-axis are reported the tested strains for biofilm production: one standard (*S. aureus* ATCC 6538) and ten MRSA clinical isolates (progressively numbered); in the y axis, the OD_570_ values of the tested bacterial strains. The bars represent the means ± SD of three independent experiments (SD = standard deviation).

For biofilm quantification, based on optical density (O.D.), determined at λ = 570 nm, the bacterial strains were classified as non-adherent, weakly adherent and strongly adherent [30]. *S. aureus* ATCC 6538, a well-known biofilm-forming strain [31], was the major producer (O.D. = 0.883). Among MRSA strains, 30% were non-adherent, 60% weakly adherent and 10% strongly adherent (Figure 2A,B). For the subsequent experiments, the standard strain and a strongly adherent MRSA strain (MRSA 8) were used.

### 2.6. MBIC, MBEC and MTT Reduction Assay

**Figure 3:**
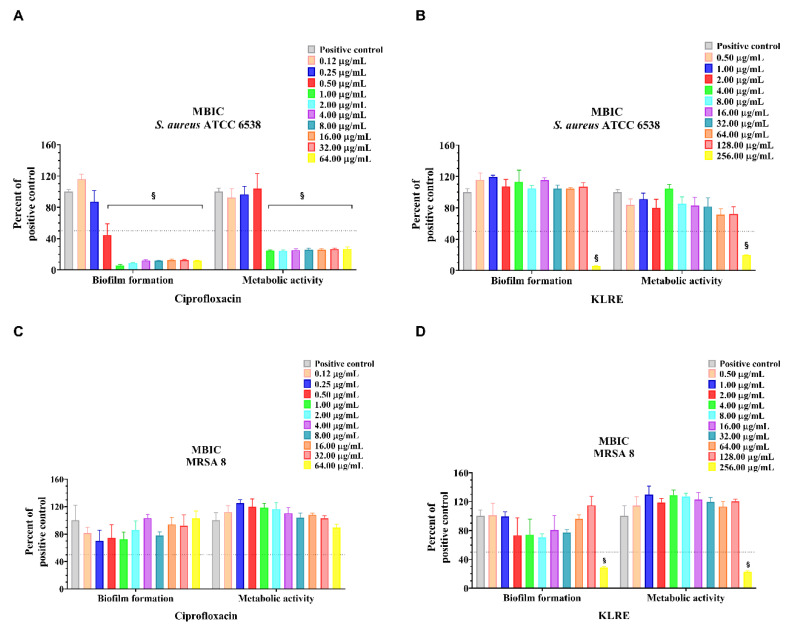
Minimal biofilm inhibitory concentration (MBIC) of ciprofloxacin and *K. lappacea* root extract (KLRE) on *S. aureus* ATCC 6538 and MRSA 8. *S. aureus* ATCC 6538 and MRSA 8 were exposed to different concentrations of ciprofloxacin (**A**,**C**), ranging from 0.12 to 64.00 μg/mL or KLRE (**B**,**D**), ranging from 0.50 to 256.00 μg/mL, for 24 h. In the x-axis are reported the biofilm formation and metabolic activity of untreated (positive control) and ciprofloxacin/KLRE treated bacterial strains. The values are expressed as a percentage of positive control. The bars represent the means ± SD of three independent experiments (SD = standard deviation). Statistically significant differences, determined by two-way analysis of variance ANOVA, are indicated ^§^ *p* ≤ 0.0001 versus positive control.

**Figure 4:**
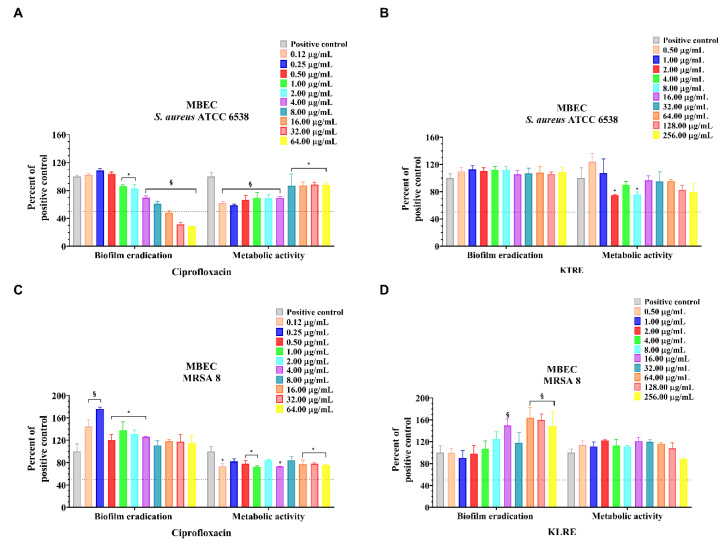
Minimal biofilm eradication concentration (MBEC) of ciprofloxacin and *K. lappacea* root extract (KLRE) on *S. aureus* ATCC 6538 and MRSA 8. *S. aureus* ATCC 6538 and MRSA 8 were exposed to different concentrations of ciprofloxacin (**A**,**C**), ranging from 0.12 to 64.00 μg/mL or KLRE (**B**,**D**), ranging from 0.50 to 256.00 μg/mL, for 24 h. In the x-axis are reported the biofilm eradication and metabolic activity of untreated (positive control) and ciprofloxacin/KLRE treated bacterial strains. The values are expressed as a percentage of positive control. The bars represent the means ± SD of three independent experiments (SD = standard deviation). Statistically significant differences, determined by two-way analysis of variance ANOVA, are indicated: * *p* ≤ 0.05, ^§^ *p* ≤ 0.0001 versus positive control.

The Minimal Biofilm Inhibition Concentration 50% (MBIC_50_) and the Minimal biofilm eradication concentration 50% (MBEC_50_) represented the lowest concentration of antimicrobial able to determine ≥50% of biofilm inhibition or eradication, respectively. The strains were treated with KLRE, in concentrations ranging from 256.00 to 0.50 µg/mL, to evaluate the ability to inhibit or eradicate bacterial biofilm (Figure 3 and Figure 4, panels B and D). The activity of the natural extract was compared to the standard antibiotic ciprofloxacin (Figure 3 and Figure 4, panels A and C). KLRE was able to inhibit biofilm formation and metabolic activity of clinical and standard strains at 256.00 µg/mL (Figure 3, panels B and D). Conversely, it failed to both eradicate the preformed biofilm and affect the metabolic activity, over the entire tested concentration range (Figure 4, panels B and D). The inhibitory dose 50% (ID_50_) MBIC and ID_50_ MBEC represented the lowest concentration of substance able to reduce ≥50% bacterial metabolic activity in MBIC and MBEC assays, respectively (Table 2). Interestingly, the highest tested dose of KLRE (256.00 µg/mL) was able to reduce both the biofilm formation and the metabolic activity of the MRSA strain, whereas ciprofloxacin did not show any inhibitory effect (>64.00 µg/mL). However, as mentioned before, KLRE as well as ciprofloxacin did not destroy the biofilm formed by the clinical strain during the 24 h of incubation (Table 2). Concerning the standard strain, already at 1.00 and 16.00 µg/mL, the antibiotic determined a reduction in the biofilm formation and preformed biofilm, respectively. However, it was not able to affect the metabolic activity of the bacteria inside the preformed biofilm, at all the tested concentrations (Table 2).

### 2.7. Effect of KLRE and Ciprofloxacin on Standard and Clinical S. aureus Strains: Growth Curves

**Figure 5:**
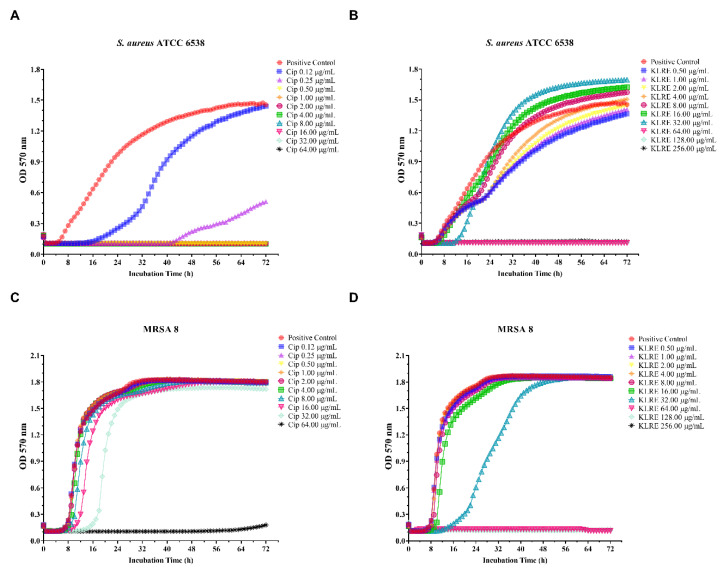
Growth curves of standard *S. aureus* ATCC 6538 and MRSA 8 exposed to different concentrations of *Krameria lappacea* root extract (KLRE) or ciprofloxacin, for 72 h. Growth trend of *S. aureus* ATCC 6538 (**A**) and MRSA 8 (**C**) untreated (positive control) and treated with increased concentrations of ciprofloxacin (Cip) ranging from 0.12 to 64.00 μg/mL. Growth trend of *S. aureus* ATCC 6538 (**B**) and MRSA 8 (**D**) untreated (positive control) and treated with increased concentrations of KLRE, ranging from 0.50 to 256.00 μg/mL. In the x-axis is reported the incubation time; in the y axis, the OD_570_ values of the two tested bacterial strains.

The effect of KLRE and ciprofloxacin on the growth of *S. aureus* strains is reported in Figure 5. After 72 h of incubation, *S. aureus* ATCC 6538 and MRSA 8 positive controls showed rapid growth, with the highest OD_600_ values of about 1.500 and 1.800, respectively. It is worth noting that KLRE completely inhibited the growth of the standard and clinical strains at a concentration of 64.00 µg/mL. Furthermore, at 32.00 µg/mL, KLRE was able to prevent the growth of the MRSA 8 strain more efficiently than the standard ones (Figure 5, panels B and D). The trend of MRSA growth curves clearly showed the resistance of the clinical isolate to the ciprofloxacin, which arrested its growth only at the highest dose. Conversely, the antibiotic significantly reduced the growth of the standard strain already at 0.12 µg/mL, totally inhibiting it at 0.50 µg/mL (Figure 5, panels A and C).

### 2.8. Effect of KLRE on the Adhesion of S. aureus to Human Lung A549 Cell Line

**Figure 6:**
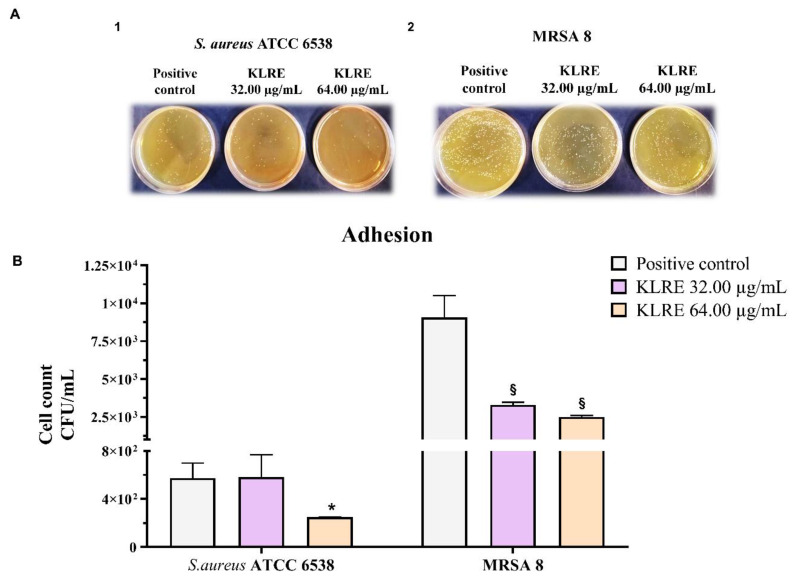
Effect of *K. lappacea* root extract (KLRE) on the adhesion of *S. aureus* ATCC 6538 and MRSA 8 to human lung A549 cell line. (**A**1, **A**2). Representative photographs of plate bacterial count. (**B**). Quantitative analysis of bacterial adhesion to A549 cells. Light grey histograms: A549 cells infected with *S. aureus* ATCC 6538 or MRSA 8 strains (positive controls). Violet histograms: A549 cells infected with standard or clinical strains and simultaneously treated with 32.00 μg/mL of KLRE. Pink histograms: A549 cells infected with standard or clinical strains and simultaneously treated with 64.00 μg/mL of KLRE. The values are expressed as colony-forming unit mL^−1^ (CFU/mL). The bars represent means ± SD of three independent experiments performed in triplicate (SD = standard deviation). Statistically significant differences, determined by one-way analysis of variance ANOVA, are indicated: * *p* ≤ 0.05, § *p* ≤ 0.0001 versus positive control.

The adhesion of *S. aureus* strains to the surface of A549 lung epithelial cells was determined through the plate count agar method (Figure 6, panels A1 and A2). Considering that the MBC values of KLRE on standard and clinical strains were 64.00 µg/mL and 128.00 µg/mL, respectively (Table 1), we chose to treat the infected A549 cells with the subtoxic concentrations of the extract for both strains (32.00 µg/mL and 64.00 µg/mL). The results showed an anti-adhesive effect of KLRE, which was more evident at 64.00 µg/mL. Indeed, this concentration determined a cell count reduction of about 50% and 70% for standard and clinical strains, respectively, compared to the untreated controls (Figure 6, panel B).

### 2.9. Effect of KLRE on the Invasion of S. aureus to Human Lung A549 Cell Line

**Figure 7:**
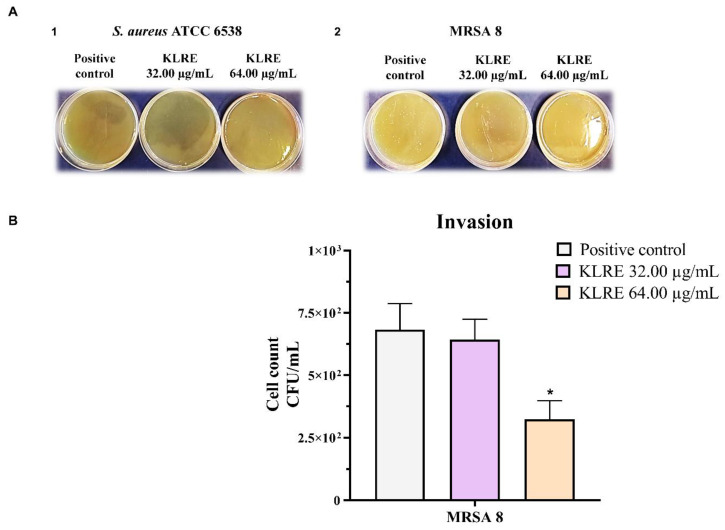
Effect of *K. lappacea* root extract (KLRE) on the invasion of *S. aureus* ATCC 6538 and MRSA 8 to human lung A549 cell line. (**A**1, **A**2). Representative photographs of plate bacterial count. (**B**). Quantitative analysis of bacterial invasion to A549 cells. Light grey histogram: A549 cells infected with MRSA 8 strain (positive control). Violet histogram: A549 cells infected with MRSA 8 and simultaneously treated with 32.00 μg/mL of KLRE. Pink histogram: A549 cells infected with MRSA 8 and simultaneously treated with 64.00 μg/mL of KLRE. The values are expressed as colony-forming unit mL^−1^ (CFU/mL). The bars represent means ± SD of three independent experiments performed in triplicate (SD = standard deviation). Statistically significant differences, determined by one-way analysis of variance (ANOVA), are indicated: * *p* ≤ 0.05, *p* ≤ 0.0001 versus positive control.

The ability of adherent *S. aureus* strains to internalize into A549 cells was determined by count bacteria from lysed eukaryotic cells on agar plates. *S. aureus* ATCC 6538 was not able to pass through the A549 cell membrane. This was confirmed by the absence of bacterial growth on agar plates (Figure 7, panel A1). Conversely, MRSA 8 was able to internalize (Figure 7, panel A2). Similar to adhesion assay, KLRE at 64.00 µg/mL reduced the cell count by about 50% with respect to untreated control (Figure 7, panel B).

### 2.10. Effect of KLRE on the Adhesion of S. aureus to Human Lung A549 Cell Line: Gram Staining

**Figure 8:**
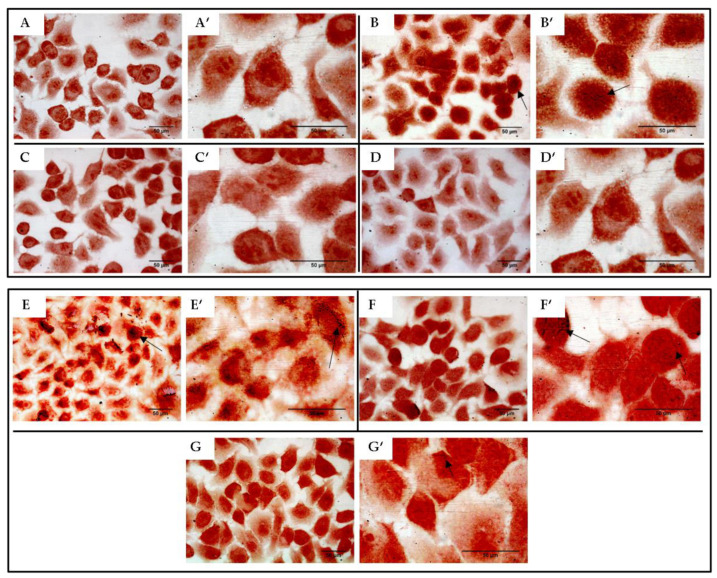
*S. aureus* ATCC 6538 (panels A-D) and MRSA 8 (panels E-G) adhesion to human lung A549 cells visualized using Gram staining (40× magnification A-G; 100X A’-G’). (**A**,**A’**): uninfected cells (negative control); (**B**,**B’**): A549 cells infected with *S. aureus* ATCC 6538 (positive control); (**C**,**C’**): A549 cells infected with *S. aureus* ATCC 6538 and simultaneously treated with 32.00 μg/mL of *K. lappacea* root extract (KLRE); (**D**,**D’**): A549 cells infected with *S. aureus* ATCC 6538 and simultaneously treated with 64.00 μg/mL of KLRE; (**E**,**E’**): A549 cells infected with MRSA 8 (positive control); (**F**,**F’**): A549 cells infected with MRSA 8 and simultaneously treated with 32.00 μg/mL of KLRE; (**G**,**G’**): A549 cells infected with MRSA 8 and simultaneously treated with 64.00 μg/mL of KLRE. Adherent colonies of *S. aureus* to A549 cell surface (black arrows).

To further investigate the adhesion ability of the two *S. aureus* strains on human lung A549 cells, we performed a Gram staining (Figure 8, panels AA’–GG’). Following the staining, *S. aureus* can be easily recognized as violet cocci distributed on the A549 cell surface (Figure 8, black arrows). The clinical isolate (Figure 8, panels EE’) showed a higher adhesion capacity compared to the standard strain (Figure 8, panels BB’). The treatment with KLRE was able to prevent the adhesion of both standard (Figure 8, panels CC’–DD’) and clinical (Figure 8, panels FF’–GG’) strains to A549 cells, in a dose-dependent manner. Because of the high adhesion, the effect of the extract was particularly evident on the MRSA strain, which visibly reduced the density of adherent bacteria compared to the respective positive control, mainly at 64.00 μg/mL (Figure 8, panels GG’ vs. panels EE’).

### 2.11. Detection of Flavonoids and Proanthocyanidins by UHPLC-HRMS

**Figure 9:**
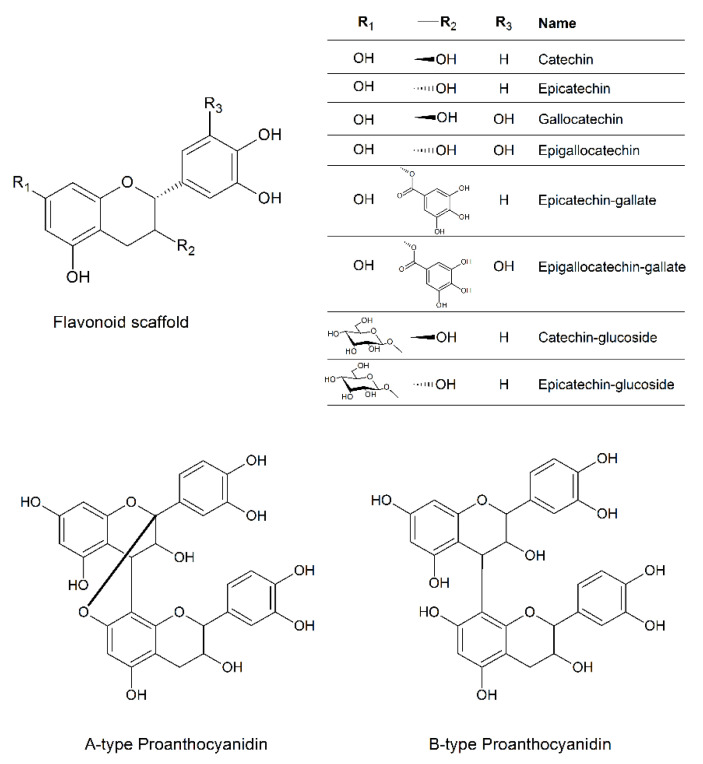
Chemical structure of the detected flavonoid and proanthocyanidin compounds in KLRE.

The content of flavonoids and related proanthocyanidins in the natural extract was determined by using a methanolic solution in order to find a wide range of flavonoids and related compounds. We also took advantage of the performance of the nano UHPLC system in terms of effective separation of the components. Finally, the optimal selectivity and the high resolution due to the Q-Orbitrap analyzer of the ESI-MS detection allowed us to unambiguously assign some of the detected charged species to well-known flavonoids and proanthocyanidins. The high accuracy of the MS detection is strictly linked to the detection of the analytes as charged ions. In the flavonoid scaffold (Figure 9), only the hydroxyl groups can be ionized and the deprotonation process depends on the relative pKa values. Therefore, the formation of negatively charged species both in solution and within the ionization spray of the ESI source led us to analyze the charged species in negative-ion mode. Table S2 reports all the detected flavonoids species along with the chromatographic and MS details. Some of them are isobaric species (i.e., catechin/epicatechin and related compounds). However, the same m/z value notwithstanding, their structural differences account for the different RT values that the isomer flavonoids have. The gallate and glucoside derivatives of several flavonoids were identified as well. Finally, both type-A and type-B proanthocyanidins dimer form (Figure 9) were also detected.

## 3. Discussion

Pneumonia is the leading cause of hospitalization and death worldwide [32]. Most HAPs are associated with the use of medical devices, particularly those supporting the spontaneous breathing of the patients. Indeed, the use of mechanical ventilators represents a serious risk factor for VAP, because it predisposes the individuals to the development of opportunistic infections caused by potentially drug-resistant organisms [33]. Among these pathogens, MRSA is one of the most common etiological agents involved in VAP [34,35,36]. Accordingly, MRSA-related VAPs are responsible for the high mortality rates among hospitalized patients admitted to the intensive care units (ICU) [37]. In this regard, in the current epidemic era, we are assisting a dramatic increase in patients who need mechanical ventilation and a long hospitalization period. These injurious events significantly contribute to the increment of the cases of MRSA-related VAPs. Recent studies proved that patients with a severe pulmonary illness caused by severe acute respiratory syndrome coronavirus 2 (SARS-CoV2) -infection can incur an increased risk for bacterial superinfections mainly represented by *S. aureus* related-VAP [38,39].

The drastic consequences of the MRSA-infections could be substantially attributed to the concomitant action of the different virulence factors, by which the organism can damage the host (Figure 1). Moreover, the resistance to a wide range of antimicrobial agents strongly compromises the success of therapy, leading to a worsening of clinical conditions of the MRSA-infected patients.

In this scenario, the role of natural extracts assumes increasing importance since they constitute a natural matrix rich in biologically active compounds. In this regard, data reporting antibacterial activity of Rathany extract against *S. aureus* led us to further explore its ability to prevent MRSA infection and invasion. Accordingly, in the present study, we demonstrated that KLRE efficiently counteracted the growth of all tested *S. aureus* strains (Table 1). Besides the growth-inhibition activity, KLRE induced the bacterial death of both standard and MRSA strains, although in the highest tested doses (256 µg/mL). Nevertheless, according to Saraiva et al., by acting at concentrations under 500 µg/mL, KLRE proved to be an effective antimicrobial agent [40]. Our results are supported by a study investigating the antibacterial effect of two different KLREs: ethyl acetate and methanol extracts. Specifically, the authors showed higher activity of ethyl acetate extract, which inhibited the growth of a large variety of Gram-positive strains, including *S. aureus* (MIC of 20 µg/mL). In this case, the low MIC value obtained could be due to the extraction method, responsible for the different chemical compositions of the extract [28].

Notably, recent work demonstrated the cytotoxic effects of different *K. lappacea* extracts, including the ethanolic extract, the object of the present study, on human breast cancer MCF-7 cell line. The authors showed a cytotoxic activity of KLRE at the concentration of 1000 µg/mL, therefore defining it as the least effective among the tested *K. lappacea* extracts. The absence of cell cytotoxicity of the extract, at the doses at which a natural extract should be considered biologically active, may be viewed as a further property supporting its possible pharmacological applications.

As previously mentioned, the virulence of *S. aureus* relies on different factors, including the ability to develop biofilm (Figure 1C). This matrix, in which the organism takes refuge from the attack of the immune cells, resists the action of most antimicrobials [41]. This stratagem allows the pathogen to persist in the host also following the antibiotic therapy, provoking recurrent infections [42]. In light of these considerations, we tested the ability of KLRE to inhibit the biofilm formation (Figure 3) or eradicate (Figure 4) the biofilm previously formed by two *S. aureus* strains: the standard and the MRSA 8 (strongly adherent). Furthermore, by the tetrazolium salt 3-[4,5-dimethylthiazol-2-yl]-2,5-diphenyltetrazolium bromide (MTT) assay, the capacity of the extract to affect the viability of the two bacteria inside the biofilm (sessile bacteria) was also evaluated. Interestingly, the treatment with 256.00 µg/mL of KLRE showed an inhibitory effect on both the biofilm formation as well as the metabolic activity of standard and MRSA strains. These findings are consistent with other studies that have proved the efficacy of natural products in inhibiting the development of biofilm [42,43,44]. Concerning that, in our previous paper, we demonstrated the suppressive action of walnut (*Juglans regia*) pellicle extract on growth, biofilm formation, and cell viability of coagulase-negative staphylococci (CoNS) [21]. However, KLRE did not affect the preformed biofilm and the viability of the sessile bacteria of both strains. Regarding the reference antibiotic ciprofloxacin, it was able to dissolve the biofilm previously formed by standard strain, but it did not reduce its viability. Besides, this antimicrobial agent was unable to either eradicate the biofilm formed by MRSA strain and affect the viability of sessile bacteria. The failure of KLRE and ciprofloxacin in disrupting the preformed biofilm reflects the refractory nature of this matrix, which is from 10- to 1000-fold more resistant to antimicrobials than planktonic bacteria [45,46].

To better investigate the antimicrobial and anti-invasive action of KLRE, we analyzed the growth curves of both standard and MRSA strains and their adhesion and invasion ability on human lung A549 cells. It is interesting to highlight that KLRE more efficiently reduced the growth of MRSA strain with respect to the reference antibiotic (Figure 5). Conversely, despite the high susceptibility of the standard strain to ciprofloxacin, which reduced its growth already at 0.25 µg/mL, this strain showed a certain resistance to KLRE, confirming the MIC values. Moreover, the in vitro assay and the Gram staining demonstrated a stronger inclination of MRSA strain to adhere to the lung cells compared to the standard strain (Figure 6 and Figure 8). Nonetheless, KLRE significantly reduced the number of adherent bacteria to A549 cells, mainly at 64.00 µg/mL. Concerning the invasive potential, according to its overall lower aggressiveness, the standard strain was not able to penetrate the lung cells. The multidrug-resistant MRSA strain, instead, showed an elevated capacity to cross through the A549 cell membrane, revealing its highly invasive potential. Once again, the treatment with KLRE significantly reduced the number of bacteria inside the A549 cells, in a dose-dependent manner. It is well established that the adhesins play a key role in the attachment and invasion of *S. aureus* to epithelial cells. The binding of these bacterial surface proteins, including the fibronectin-binding proteins, with specific cellular receptors or with the components of the extracellular matrix (e.g., fibronectin) activates a signalling cascade that culminates with the internalization of the bacterium into the host cell (Figure 1B) [4,5,47]. Therefore, KLRE may interfere with this process, exerting an anti-adhesive action. On the other hand, Scholz and Rimpler detected in the extract the proanthocyanidins [27]. Scientific evidence highlighted the antibacterial [48] and anti-biofilm [49] potential of these compounds against MRSA strains. Further studies proved an inhibitory effect of the proanthocyanidins on the adhesion and invasion of different bacterial species [50,51]. Interestingly, Hui et al. demonstrated that the proanthocyanidins are able to prevent the adhesion of *S. aureus* on biomaterials [52]. According to these findings, we analyzed our extract by UHPLC-HRMS to detect both flavonoids and related proanthocyanidins [53]. As expected, several flavonoid compounds and type-A and -B proanthocyanidins were detected. Therefore, the accurate MS-based analysis confirmed the published data about the composition of KLRE. Then, these compounds reasonably contribute to the activities shown by this natural extract, even though the role of other natural constituents cannot be excluded.

## 4. Materials and Methods

### 4.1. Chemicals

All the solvents, reference compounds and chemicals were of reagent grade and purchased from Sigma-Aldrich (Milan, Italy).

### 4.2. Plant Material and Preparation of the Extract

**Figure 10:**
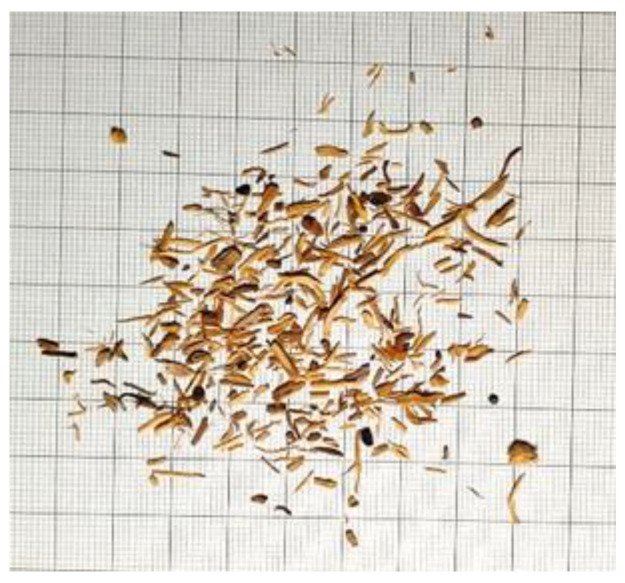
*Krameria lappacea* roots.

*Krameria lappacea* (KL) roots (Figure 10) were purchased from the Herbal shop of Dr. Milardo Fabio, lot number: E598-02/07/23, production year 2019. The extract was obtained by maceration of 10 g of KL grounded roots in 100 mL of ethanol, for 72 h, at room temperature, under constant shaking. The extraction was repeated three times. Afterwards, the three aliquots were reunited, filtered through a Whatman^®^ Grade 1 filter paper (Whatman, UK) and evaporated to dryness at 40 °C with a rotatory evaporator (Stuart RE300, Cole-Parmer Ltd^®^, Stone, UK), obtaining 0.275 g of dry extract.

### 4.3. Bacterial Strains and Human Cells

Ten MRSA strains, isolated from respiratory infections, were from the bacterial library of the Department of Biomedical and Biotechnological Sciences, Section of Microbiology. The reference strains, *S. aureus* ATCC 6538, *S. aureus* ATCC 43300 and *S. aureus* ATCC 29213 and A549 human lung epithelial cells (ATCC^®^ CCL-185^TM^) were purchased from LGC Limited (Teddington, Middlesex, UK).

### 4.4. Determination of S. aureus Strains Susceptibility to Cefoxitin and Oxacillin by Disc-Diffusion Test

The susceptibility of *S. aureus* strains to 30 µg cefoxitin (Becton Dickinson, Franklin Lakes, NJ, USA) and 1 µg oxacillin (Becton Dickinson, Franklin Lakes, NJ, USA) was determined by the disc diffusion test, according to the CLSI guidelines [29]. The suspensions 0.5 McFarland standard, equivalent to 1.5 × 10^8^ colony-forming units/mL (CFU/mL), were spread with a sterile swab on Mueller-Hinton agar (MHA) plates (Oxoid, Milan, Italy) and the disks were applied to the surface. After incubation for 24 h at 35 °C, the diameter inhibition zone was determined. *S. aureus* ATCC 6538 was used as a reference strain (negative control).

### 4.5. Detection of Macrolide-Lincosamide-Streptogramin B Phenotypes

Macrolide-lincosamide-streptogramin B (MLS_B_) phenotypes were determined by the double-disk diffusion test on MHA, as previously described [54].

### 4.6. Detection of Penicillin-Binding Protein 2a (PBP2a)

*S. aureus* strains were screened for the presence of PBP2a. The protein was detected through the Oxoid Penicillin Binding Protein (PBP2a) Latex Agglutination Test (Oxoid, Milan, Italy). The assay was performed according to the manufacturer’s guidelines. Each test included a positive control (*S. aureus* ATCC 43300) and negative control (*S. aureus* ATCC 29213).

### 4.7. Antibacterial Activity of Krameria Lappacea Root Extract (KLRE)

The antibacterial activity of KLRE was tested by the microdilution method, according to the standard procedures of the CLSI [29]. The dry extract (0.256 g) was solubilized in DMSO and diluted in the 1:100 ratio in BBLTM Cation-adjusted Mueller Hinton II Broth (CAMHB) (Becton DickinsonFranklin Lakes, NJ, USA). After filtration of the stock solution with a 0.22 μm filter (Abluo^®^, GVS S.p.a., Bologna, Italy), serial twofold dilutions were made in concentrations ranging from 0.50 to 256.00 μg/mL in sterile 96-well microplates (Corning, New York, NY, USA) containing CAMHB. Isolated colonies on MHA plates were suspended in saline solution and the turbidity adjusted to a 0.5 McFarland standard. The suspension turbidity was evaluated through a spectrophotometer at λ = 600 nm (Bio-Tek Synergy HT Microplate Reader, Bio-Tek Instruments, Winooski, VT, USA). After a dilution in the 1:100 ratio, bacterial suspensions were added to each well obtaining a final concentration of 5 × 10^5^ CFU/mL. The MIC was the lowest concentration at which there was no visible growth after incubation at 37 °C for 24 h. The MBC was the lowest concentration that killed 99.9% of the bacterial population [29]. Ciprofloxacin was used as an antibacterial positive control, in concentrations ranging from 64.00 to 0.12 µg/mL. Besides, the MBC/MIC ratio was calculated in order to determine if the compounds had a bacteriostatic (MBC/MIC ratio > 4.00) or bactericidal (MBC/MIC ratio ≤ 4.00) activity [55]. Each test included negative sterility control and positive growth control. Results are expressed as the mean of three experiments.

### 4.8. Biofilm Quantification Assay

Biofilm formation was determined in flat-bottom 96-well polystyrene microplates (Corning, New York, NY, USA) according to Stepanović et al. with some modifications [56]. A total of 200 μL of bacterial suspension 0.5 McFarland (1.5 × 10^8^ CFU/mL) in Tryptic Soy Broth medium (Becton Dickinson, Franklin Lakes, NJ, USA) with 1% (*w*/*v*) D-(+)-glucose (TSBG) was inoculated in wells and incubated at 37 °C for 24 h. After the incubation period, the wells were discharged and washed three times with 200 μL of phosphate-buffered saline (PBS). 200 μL of 1% (*v*/*v*) crystal violet (CV) (Merck, Damm, Germany) was added per well for 15 min and after the wells were discharged and washed three times with 200 μL of PBS. The microplates were air-dried and the biofilm-bound CV was dissolved adding 200 μL of 96% (*v*/*v*) ethanol per well. O.D. was determined at λ = 570 nm [56,57,58]. Bacterial strains were categorized as non-adherent (O.D. ≤ 0.120), weakly adherent (O.D. > 0.120) and strongly adherent (O.D. > 0.240) [30]. Results are expressed as the mean of three experiments.

### 4.9. Minimum Biofilm Inhibitory Concentration Assay

MBIC was the lowest concentration of an antimicrobial agent that prevents biofilm formation. To determine the MBIC of KLRE, 200 μL of serial dilutions in concentrations ranging from 256.00 to 0.50 μg/mL in TSBG were added in 96-well microplates. The final concentration per well of bacterial strains was 1.5 × 10^8^ CFU/mL. After the incubation at 37 °C for 24 h, the wells were discharged and washed three times with 200 μL of PBS. In total, 200 μL of 1% (*v*/*v*) CV was added in each well for 15 min. The wells were discharged and washed three times with 200 μL of PBS. Finally, the microplates were air-dried and the biofilm-bound CV was dissolved with 200 μL of 96% (*v*/*v*) ethanol. O.D. was measured at λ = 570 nm [56,57,58,59]. Results are expressed as the mean of three experiments.

### 4.10. Minimum Biofilm Eradication Concentration Assay

MBEC was the lowest concentration of an antimicrobial agent which can damage the biofilm structure. For MBEC assay, 200 μL of a 0.5 McFarland bacterial suspension in TSBG was added in each well and incubated at 37 °C for 24 h. After the incubation, the wells were discharged and washed three times with 200 μL of PBS to remove slightly adherent planktonic cells. An amount of 200 μL of KLRE in concentrations ranging from 256.00 to 0.50 μg/mL in TSBG were added to the microplates. After the incubation at 37 °C for 24 h, the wells were discharged and washed three times with 200 μL of PBS. Bacterial biofilm was stained with 200 μL of 1% (*v*/*v*) CV for 15 min. Afterwards, the wells were discharged and washed three times with 200 μL of PBS. Finally, the microplates were air-dried and CV was dissolved with 200 μL of 96% (*v*/*v*) ethanol. O.D. was measured at λ = 570 nm [56,57,58,59]. Results are expressed as the mean of three experiments.

### 4.11. MTT Reduction Assay

The viability of bacteria inside biofilm was determined through MTT assay. Metabolically active cells metabolize the yellow tetrazole into insoluble purple formazan product. Accordingly, this method allowed us to assess biofilm susceptibility to natural extract compared to standard drug ciprofloxacin. MBIC and MBEC assays were performed as previously described. At the end of the two treatments, the wells were discharged and washed three times with 200 μL of PBS. The MTT solution (0.5 mg/mL in PBS) was added to the microplates. After incubation for 2 h at 37 °C, 100 μL of dimethyl sulfoxide were added to each well to dissolve the purple formazan inside biofilms. The microplates were incubated for 20 min in the dark, with agitation, at room temperature. O.D. was determined at λ = 570 nm [60].

### 4.12. Growth Curve

The bacterial overnight cultures were diluted into CAMHB containing different concentrations of KLRE (0.50–256.00 μg/mL) and ciprofloxacin (0.12–64.00 µg/mL). The final concentration of bacteria per well was approximately 5 × 10^5^ CFU/mL. The 96 well microplates were incubated in the Bio-Tek reader at 37 °C for 72 h. Measurements were made at λ = 600 nm, every 60 min, with continuous orbital shaking (50 *g*).

### 4.13. Cell Culture

A549 human lung epithelial cells (ATCC^®^ CCL-185^TM^) were cultured in Dulbecco’s Modified Eagle’s Medium (DMEM) (Sigma-Aldrich, Milan, Italy) supplemented with 10% (*v*/*v*) heat-inactivated fetal bovine serum (FBS), 2 mM glutamine, 5 mg/mL penicillin and 100 mg/mL streptomycin. The cells were incubated at 37 °C, 5% CO_2_ atmosphere and 95% relative humidity. Cells were passaged once a week after trypsinization and replaced with a new medium twice weekly.

### 4.14. Bacterial Invasion and Adhesion Assay

Bacterial invasion and adhesion assays were performed according to Cue et al., with some modifications [61]. A549 cells were seeded in 24 well microplates (Corning) at a density of 2 × 10^5^ cells/well. A microplate containing 12 mm diameter coverslips (Thermo Scientific Menzel) was used for microscopic analysis. Confluent monolayers were infected with 500 µL of 1 × 10^5^ CFU/mL bacterial suspension in DMEM. The microplates were incubated at 37 °C, for 2 h in a 5% CO_2_ atmosphere. Following incubation, infected monolayers were washed three times with 1 mL of PBS. DMEM—FBS containing 5 mg/mL penicillin and 100 mg/mL gentamicin was added in each well to kill extracellular and non-adherent bacteria. After 2 h of incubation at 37 °C, cell monolayers were washed three times with PBS, dispersed in 200 µL of 0.25% trypsin—1 mM EDTA and lysed by adding 800 µL of sterile distilled water for 1 h. The number of internalized bacterial cells was determined by plating diluted lysates in Mueller Hinton agar (MHA) plates (Oxoid, Milan, Italy) and expressed in CFU/mL. For bacterial adhesion assay, the culture medium was removed from cell monolayers after the invasion period and three washes with PBS were carried out to remove non-adherent bacteria. The cells were dispersed, lysed and plated on MHA. The proportion of adherent bacteria was calculated by subtracting from the total CFU/mL (intracellular plus extracellular) the number of internalized bacteria. Experiments were performed in triplicate.

### 4.15. Gram Staining

Cell-adherent bacteria were visualized by Gram staining [62,63], with some modifications. The coverslips (12 mm diameter) inside a 12-well microplate were stained with 1 mL of 1% (*v*/*v*) CV for 2 min. The wells were discharged and washed two times with 1 mL of distilled water. Afterwards, 1 mL of 1% (*v*/*v*) Lugol’s iodine solution was added to each well for 2 min. The wells were discharged and washed two times with 1 mL of distilled water. A few drops of an acetone-ethanol mixture (50:50 *v*/*v*) were added to decolorize glasses and, immediately, three washing with 1 mL of distilled water were carried out. Finally, 1 mL of 1% (*v*/*v*) safranine solution was added to each well and maintained for 3 min. The glasses were washed two times with 1 mL of distilled water and air-dried. Microscopic analysis was performed using the 40× and 100× oil immersion objective (plus 10× ocular), using Leica DMRB Fluorescence Microscope. *S. aureus* strains adhering to A549 cells were visible as violet cocci on a pale red background. Digital images were acquired by a computer-assisted digital camera (Leica DFC 320, 3.3 Megapixel; Software: Leica Application Suite 2.8.1).

### 4.16. Flavonoid Extraction and UHPLC-HRMS Analysis

KLRE (10 mg) was treated with 1.5 mL of methanol: water 70:30. The mixture was sonicated for 10 min (60 kHz and 30 W) and centrifuged at 10,000× *g* for 10 min. The supernatant was filtered (0.2 µm) and diluted in the 1:100 ratio with 20% (*v*/*v*) acetonitrile in water containing formic acid (FA) 0.1% (*v*/*v*) before the LC-MS analysis.

The qualitative analysis of flavonoids and pro-anthocyanidins was performed on a hybrid quadrupole-Orbitrap mass spectrometer (Q-Exactive, Thermo Scientific, Waltham, MA, USA) coupled to Ultimate 3000 HPLC RSLCnano system (Dionex Thermo Scientific, Waltham, MA, USA) through an EASY-Spray source (Thermo Scientific, Waltham, MA, USA), by using a setting previously reported [64] with few modifications. Briefly, capillary temperature and voltage were 300 °C and 2 kV, respectively. The MS acquisition was performed in negative ion mode (70,000 resolution, scan range 500 to 2000 m/z, maximum injection time 50 ms, AGC target 1 × 10^6^) and MS/MS mode (17,500 resolution, scan range 200 to 2000 m/z, maximum injection time 50 ms, AGC target 1 × 10^5^). The chromatographic separation of compounds was performed on EASY-Spray PepMap^®^ C18 column (75 μm × 150 mm, 3 μm particle size, 300 Å pore size) at a flow rate of 0.3 μL/min with solvent A (water with 0.1% FA) and solvent B (80% acetonitrile, 0.1% FA in water). All the compounds were eluted by using a linear gradient from 20% to 80% of eluent B. Peak detection for the evaluation of flavonoids and proanthocyanidins was carried out using the extracted ion chromatogram (XIC) related to the single-charged species detected for each compound.

### 4.17. Statistical Analysis

Data are expressed as mean ± standard deviation (±SD). We evaluated the statistical significance of our data by applying the one or two-way ANOVA analysis, to assess the significance of at least three sample groups from three different experiments (i.e., biological and technical triplicates). Data analysis and graphical representations were performed by using GraphPad Prism 8 software (GraphPad, San Diego, CA, USA).

## 5. Conclusions

The investigation of the effects of KLRE on *S. aureus* pathophysiology adds a small piece to the current knowledge on the biological activity of this extract, shedding light on an interesting but still poorly characterized plant. Furthermore, the promising antimicrobial properties of KLRE against one of the most virulent bacteria and the possible correlation of these effects with the chemical composition of the extract certainly support further studies aimed to better elucidate the mechanism of action through which the KLRE-chemical constituents act.

## Figures and Tables

**Table 1:** Minimal Inhibitory Concentration (MIC), Minimal Bactericidal Concentration (MBC) and MBC/MIC ratio of *Krameria lappacea* root extract (KLRE) and ciprofloxacin against *Staphylococcus aureus* strains.

	KLRE Range [256.00-0.50 µg/mL]				Ciprofloxacin Range [64.00-0.12 µg/mL]				
Bacterial strains ^a^	MIC ^b^	MBC ^c^	MBC/MIC ^d^	EFF. ^e^	MIC	MBC	MBC/MIC	EFF.	I.C. ^f^
*S. aureus* ATCC 6538	64.00	128.00	2	BC	0.25	0.25	1	BC	S
MRSA1	64.00	128.00	2	BC	32.00	32.00	1	BC	R
MRSA 2	64.00	256.00	4	BC	64.00	64.00	1	BC	R
MRSA 3	64.00	256.00	4	BC	64.00	64.00	1	BC	R
MRSA 4	64.00	128.00	2	BC	32.00	32.00	1	BC	R
MRSA 5	64.00	128.00	2	BC	32.00	32.00	1	BC	R
MRSA 6	64.00	256.00	4	BC	32.00	32.00	1	BC	R
MRSA 7	64.00	128.00	2	BC	64.00	64.00	1	BC	R
MRSA 8	64.00	128.00	2	BC	64.00	64.00	1	BC	R
MRSA 9	64.00	256.00	4	BC	32.00	32.00	1	BC	R
MRSA 10	64.00	128.00	2	BC	16.00	16.00	1	BC	R

^a^ Strain numbers refer to an internal directory for clinical isolates; methicillin-resistant *Staphylococcus aureus* (MRSA) strains, isolated from respiratory infections, belonged to the bacterial library of the Department of Biomedical and Biotechnological Sciences; ^b^ MIC: Minimal Inhibitory Concentration; ^c^ MBC: Minimal Bactericidal Concentration; ^d^ MBC/MIC ratio ≤ 4: bactericidal, MBC/MIC ratio >4: bacteriostatic; ^e^ BC: bactericidal, BS: bacteriostatic; ^f^ I.C.: Interpretive criteria for ciprofloxacin (CLSI M100-S30): ≤ 1 Sensitive (S); 2 Intermediate (I); ≥ 4 Resistant (R).

**Table 2:** Effect of *Krameria lappacea* root extract (KLRE) and ciprofloxacin on biofilm formation (MBIC_50_), biofilm eradication (MBEC_50_) and metabolic activity (ID_50_) of *S. aureus* strains.

	KLRE Range [256.00–0.50 µg/mL]				Ciprofloxacin Range [64.00–0.12 µg/mL]			
Bacterial Strains	MBIC_50_^a^	ID_50_ MBIC ^b^	MBEC_50_^c^	ID_50_ MBEC ^d^	MBIC_50_	ID_50_ MBIC	MBEC_50_	ID_50_ MBEC
*S. aureus* ATCC 6538	256.00	256.00	>256.00	>256.00	1.00	1.00	16.00	>64.00
MRSA 8 ^e^	256.00	256.00	>256.00	>256.00	>64.00	>64.00	>64.00	>64.00

^a^ MBIC_50_: Minimal Biofilm Inhibitory Concentration (inhibition ≥50% with respect to positive control); ^b^ ID_50_ MBIC: ≥50% reduction in metabolic activity in MBIC assay; ^c^ MBEC_50_: Minimal Biofilm Eradication Concentration (eradication ≥50% with respect to positive control); ^d^ ID_50_ MBEC: ≥50% reduction in metabolic activity in MBEC assay; ^e^ strain number refers to an internal directory for clinical isolates.

## Data Availability

The data presented in this study are available on request from the corresponding author.

## Supplementary Materials

The following are available online at https://www.mdpi.com/article/10.3390/antibiotics10040428/s1, Table S1: Susceptibility pattern of *S. aureus* strains and detection of PBP2a; Table S2: Chromatographic and spectrometric details of the flavonoid and proanthocyanidin compounds in KLRE.
