# Correction: Genovese et al. In Vitro Antibacterial, Anti-Adhesive and Anti-Biofilm Activities of *Krameria lappacea* (Dombey) Burdet & B.B. Simpson Root Extract against Methicillin-Resistant *Staphylococcus aureus* Strains. *Antibiotics* 2021, *10*, 428

**DOI:** 10.3390/antibiotics10070799

**Published:** 2021-06-30

**Authors:** Carlo Genovese, Floriana D’Angeli, Francesco Bellia, Alfio Distefano, Mariarita Spampinato, Francesco Attanasio, Daria Nicolosi, Valentina Di Salvatore, Gianna Tempera, Debora Lo Furno, Giuliana Mannino, Fabio Milardo, Giovanni Li Volti

**Affiliations:** 1 Section of Microbiology, Department of Biomedical and Biotechnological Sciences, University of Catania, via Santa Sofia 97, 95123 Catania, Italy; dnicolosi@unict.it; 2 Department of Drug and Health Sciences, University of Catania, Viale Andrea Doria 6, 95125 Catania, Italy; 3 Nacture S.r.l, Spin-Off University of Catania, via Santa Sofia 97, 95123 Catania, Italy; tempera@unict.it; 4 Section of Biochemistry, Department of Biomedical and Biotechnological Sciences, University of Catania, via Santa Sofia 97, 95123 Catania, Italy; fdangeli@unict.it (F.D.); distalfio@gmail.com (A.D.); mariaritaspampinato93@gmail.com (M.S.); livolti@unict.it (G.L.V.); 5 Department of Human Sciences and Quality of Life Promotion, San Raffaele Roma Open University, via Val Cannuta 247, 00166 Rome, Italy; 6 Institute of Crystallography, National Research Council (CNR), Via Paolo Gaifami, 18, 95126 Catania, Italy; francesco.bellia@cnr.it (F.B.); francesco.attanasio@cnr.it (F.A.); 7 Section of General and Clinical Pathology and Oncology, Department of Biomedical and Biotechnological Sciences, University of Catania, via Santa Sofia 97, 95123 Catania, Italy; valentina.disalvatore@unict.it; 8 Section of Physiology, Department of Biomedical and Biotechnological Sciences, University of Catania, via Santa Sofia 97, 95123 Catania, Italy; lofurno@unict.it (D.L.F.); giuliana.mannino@unict.it (G.M.); 9 Herbalist Shop of Dr. Milardo Fabio, via Fonte 2, 96010 Melilli, Italy; fabiomilardo@gmail.com

The authors would like to make the following corrections to the published paper [1].

There are mistakes in Figure 8 and its legend.

In the original article, Figure 8A’ was duplicated with Figure 8D’. We have replaced Figure 8D’ with a new picture.

In the first sentence of the Figure 8 legend, we inexactly indicated with the A–D letters, reported in brackets, the *S. aureus* ATCC 6538 adhesion to human lung A549 cells. However, Figure 8A indicates the uninfected cells (negative control). Therefore, we replaced A–D with B–D in the legend. 

The correct Figure 8 and its legend are as below. The authors apologize for any inconvenience caused and state that the scientific conclusions are unaffected. 

## Figures and Tables

**Figure 8:**
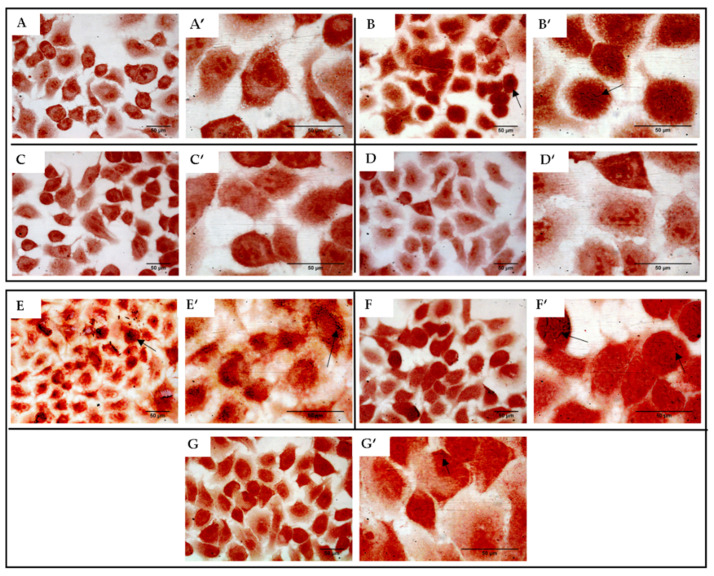
*S. aureus* ATCC 6538 (**B**–**D**) and MRSA 8 (**E**–**G**) adhesion to human lung A549 cells visualized using Gram staining. Bacterial adhesion, in the different experimental conditions, was compared to negative control (**A**). 40× magnification A–G; 100× magnification A’–G’. (**A**,**A’**): uninfected cells (negative control); (**B**,**B’**): A549 cells infected with *S. aureus* ATCC 6538 (positive control); (**C**,**C’**): A549 cells infected with *S. aureus* ATCC 6538 and simultaneously treated with 32.00 μg/mL of *K. lappacea* root extract (KLRE); (**D**,**D’**): A549 cells infected with *S. aureus* ATCC 6538 and simultaneously treated with 64.00 μg/mL of KLRE; (**E**,**E’**): A549 cells infected with MRSA 8 (positive control); (**F**,**F’**): A549 cells infected with MRSA 8 and simultaneously treated with 32.00 μg/mL of KLRE; (**G**,**G’**): A549 cells infected with MRSA 8 and simultaneously treated with 64.00 μg/mL of KLRE. Adherent colonies of *S. aureus* to A549 cell surface (black arrows).
