# Supplementary material for: In Vitro Antibacterial, Anti-Adhesive and Anti-Biofilm Activities of Krameria lappacea (Dombey) Burdet & B.B. Simpson Root Extract against Methicillin-Resistant Staphylococcus aureus Strains
Source: Antibiotics (Basel). 2021 Apr 13;10(4):428. doi: 10.3390/antibiotics10040428 (PMC8069196; doi:10.3390/antibiotics10040428)
Supplement: Supplementary file 1 [file antibiotics-10-00428-s001.pdf]

Table S1. Susceptibility pattern of *S. aureus* strains and detection of PBP2a.

Bacterial strains [a]	CXT [b] (mm)	I.C.CXT [c]	OXA [d] (mm)	I.C.OXA [e]	ERY [f] (mm)	I.C.ERY [g]	CLI [h] (mm)	I.C.CLI [i]	MLSB R [l]	PBP2a [m]
S. aureus ATCC 6538	31	S	13	n/a	30	S	30	S	-	n.d.
MRSA 1	17	R	0	n/a	0	R	0	R	$cMLS_B$ [n]	d.
MRSA 2	18	R	0	n/a	0	R	0	R	$cMLS_B$	d.
MRSA 3	18	R	0	n/a	0	R	0	R	$cMLS_B$	d.
MRSA 4	16	R	0	n/a	0	R	0	R	$cMLS_B$	d.
MRSA 5	14	R	0	n/a	0	R	0	R	$cMLS_B$	d.
MRSA 6	18	R	0	n/a	0	R	0	R	$cMLS_B$	d.
MRSA 7	14	R	0	n/a	0	R	0	R	$cMLS_B$	d.
MRSA 8	10	R	0	n/a	0	R	0	R	$cMLS_B$	d.
MRSA 9	11	R	0	n/a	0	R	0	R	$cMLS_B$	d.
MRSA 10	19	R	12	n/a	24	S	26	S	-	d.

[a] Strain numbers refer to an internal directory for clinical isolates; methicillin-resistant Staphylococcus aureus (MRSA) strains, isolated from respiratory infections, belonged to the bacterial library of the Department of Biomedical and Biotechnological Sciences; [b] CXT: cefoxitin; [c] I.C.cxt: interpretive criteria of CLSI M100-S30 for cefoxitin: ≥ 22 susceptible (S), ≤ 21 resistant (R); [d] OXA: oxacillin; [e] I.C.OXA: interpretive criteria of CLSI M100-S30 for oxacillin: not applicable (n/a), since cefoxitin is used as a surrogate for disk diffusion testing; [f] ERY: erythromycin; [g] I.C.ERY: interpretive criteria of CLSI M100-S30 for erytrhomicin: ≥ 23 susceptible (S), 14-22 intermediate (I), ≤ 13 resistant (R); [h] CLI: clindamycin; [i] I.C.CLI: interpretive criteria of CLSI M100-S30 for clindamycin: ≥ 21 susceptible (S), 15-20 intermediate (I), ≤ 14 resistant (R); [l] MLSB R: macrolide–lincosamide–streptogramin B resistance; [m] PBP2a: penicillin-binding protein 2a; d: detected, n.d.: not detected; the agglutination assay included the methicillin-resistant strain *S. aureus* ATCC 43300 and the methicillin-susceptible strain *S. aureus* ATCC 29213 as positive and negative controls, respectively; [n] cMLSB: constitutive macrolide–lincosamide–streptogramin B resistance.

Table S2. Chromatographic and spectrometric details of the flavonoid and proanthocyanidin compounds in KLRE.

**Retention Time** (min)	**[M-H]⁻ theor.** (*m/z*)	**[M-H]⁻ exp.** (*m/z*)	**Molecular Formula**	**Δm** (Da)	**Tentative compound identification**
19.83, 27.66	305.066	305.067	$C_{15}H_{13}O_7$	0.001	Gallocatechin, Epigallocatechin
20.14, 22.08	289.071	289.064	$C_{15}H_{13}O_6$	-0.007	Catechin, Epicatechin
22.4	457.077	457.081	$C_{22}H_{17}O_{11}$	0.004	Gallocatechin-gallate
23.23	441.082	441.088	$C_{22}H_{17}O_{10}$	0.006	Epicatechin-gallate
23.23, 24.2, 25.6	451.124	451.134	$C_{21}H_{23}O_{11}$	0.010	Catechin-glucoside,Epicatechin-glucoside
24.5	467.119	467.134	$C_{21}H_{23}O_{12}$	0.015	Gallocatechin-glucoside
29.95	577.135	577.144	$C_{30}H_{25}O_{12}$	0.009	Catechin (PA-B)
35.65, 39.24	575.119	575.109	$C_{30}H_{23}O_{12}$	-0.010	Catechin (PA-A)
